# Highly Efficient Solar‐Light‐Driven Photodegradation of Metronidazole by Nickel Hexacyanoferrate Nanocubes Showing Enhanced Catalytic Performances

**DOI:** 10.1002/smtd.202301541

**Published:** 2024-02-17

**Authors:** Edlind Lushaj, Matteo Bordin, Kamran Akbar, Letizia Liccardo, Isabel Barroso‐Martín, Enrique Rodríguez‐Castellón, Alberto Vomiero, Elisa Moretti, Federico Polo

**Affiliations:** ^1^ Department of Molecular Sciences and Nanosystems Ca’ Foscari University of Venice Via Torino 155 Venezia 30172 Italy; ^2^ Department of Inorganic Chemistry Crystallography and Mineralogy Faculty of Sciences University of Malaga Campus de Teatinos Malaga 29071 Spain; ^3^ Division of Materials Science Department of Engineering Sciences and Mathematics Luleå University of Technology Luleå 97187 Sweden; ^4^ European Centre for Living Technology (ECLT) Ca’ Bottacin, Dorsoduro 3911, Calle Crosera Venice 30124 Italy

**Keywords:** metronidazole, nanocubes, photocatalysis, Prussian blue analogues, wastewater treatment

## Abstract

Environmental pollution is a complex problem that threatens the health and life of animal and plant ecosystems on the planet. In this respect, the scientific community faces increasingly challenging tasks in designing novel materials with beneficial properties to address this issue. This study describes a simple yet effective synthetic protocol to obtain nickel hexacyanoferrate (Ni‐HCF) nanocubes as a suitable photocatalyst, which can enable an efficient photodegradation of hazardous anthropogenic organic contaminants in water, such as antibiotics. Ni‐HCF nanocubes are fully characterized and their optical and electrochemical properties are investigated. Preliminary tests are also carried out to photocatalytically remove metronidazole (MDZ), an antibiotic that is difficult to degrade and has become a common contaminant as it is widely used to treat infections caused by anaerobic microorganisms. Under simulated solar light, Ni‐HCF displays substantial photocatalytic activity, degrading 94.3% of MDZ in 6 h. The remarkable performance of Ni‐HCF nanocubes is attributeto a higher ability to separate charge carriers and to a lower resistance toward charge transfer, as confirmed by the electrochemical characterization. These achievements highlight the possibility of combining the performance of earth‐abundant catalysts with a renewable energy source for environmental remediation, thus meeting the requirements for sustainable development.

## Graphical abstract

Water pollution is a threat to health and ecosystems. Nickel‐hexacyanoferrate (Ni‐HCF) nanocubes provide a step forward in addressing such issue, as they show excellent photocatalytic activity toward degradation of up to ≈94% of the antibiotic metronidazole under simulated solar light in 6 h. Superior charge carrier separation and low resistance make Ni‐HCF promising for sustainable and effective environmental remediation.

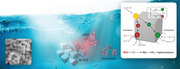

## 1 Introduction

The environmental pollution caused by human activity represents a complex problem that threatens the health and life of animal and plant ecosystems on our planet.^[^ ^1^, ^2^, ^3^ ^]^ To address this issue, the scientific community worldwide is facing more demanding challenges while designing and developing novel materials, whose properties could be beneficial to this end. In particular, when dealing with water pollution, new engineered materials have been designed, synthesized, and tested to enhance wastewater treatment processes.^[^ ^4^, ^5^ ^]^ In this context, the pollution caused by pharmaceuticals and pesticides and the consequences they pose toward wildlife and human health are of great concern.^[^ ^6^, ^7^, ^8^ ^]^ Among the pollutants present in water, antibiotics have been deeply investigated due to their abuse, their ability to cause ecological harm (e.g.,: endocrine disruption and antimicrobial resistance), and their mutagenic and carcinogenic properties.^[^ ^9^ ^]^ In this regard, 2‐(2‐methyl‐5‐nitro‐1H‐imidazol‐1‐yl) ethanol, known as metronidazole (MDZ), is an antibiotic drug widely used to treat infections caused by anaerobic microorganisms such as bacteria and protozoa.^[^ ^10^ ^]^ It has been found in municipal wastewater, groundwater, surface, and drinking water.^[^ ^11^, ^12^, ^13^ ^]^ Although very little is known about the chronic health effects and antimicrobial resistance toward this drug due to its long‐term ingestion even in very low concentrations, MDZ accumulation can cause adverse effects on aquatic ecosystems due to its intrinsic biological activity.^[^ ^14^ ^]^ Therefore, the development of novel methods to fully remove antibiotics from wastewater is of major importance. To this end, many compounds have been recently synthesized and investigated as suitable photocatalysts aimed at photodegrading MDZ, among which it is worth mentioning Bi_2_WO_6_,^[^ ^15^ ^]^ Zn_2_GeO_4_,^[^ ^16^ ^]^ Mn‐doped BiOCl,^[^ ^17^ ^]^ NiO and ZnO,^[^ ^18^, ^19^ ^]^ TiO_2_,^[^ ^20^ ^]^ and CuO.^[^ ^21^ ^]^ Other methods, such as direct photoreduction under UV irradiation,^[^ ^22^ ^]^ and Fenton and photo‐Fenton processes,^[^ ^23^ ^]^ were also explored. Although the method based on photocatalytic degradation has been regarded as a favorable strategy for decomposing antibiotics due to its notable effectiveness and cost efficiency,^[^ ^24^, ^25^ ^]^ finding a suitable photocatalyst, which is capable of efficiently removing this antibiotic under solar light irradiation, remains a challenge. It is also worth mentioning that employing only solar light as a promoter can provide many advantages,^[^ ^26^, ^27^ ^]^ such as eliminating the use of energy‐demanding UV lamps,^[^ ^20^, ^28^ ^]^ thus aligning the scientific research to the requirements of sustainable development.

Currently, Prussian Blue Analogues (PBAs) are gaining attention for their customizable frameworks, large surface areas, and abundant pore structures.^[^ ^29^, ^30^ ^]^ This stems from the fact that PBAs, having a general formula Fe_4_[Fe(CN)_6_]_3_·nH_2_O, can be easily modified and transformed into various structures. They display several advantages, namely an open 3D framework that allows the diffusion of various charge‐carrier ions in all directions, control over the composition with negligible change in the overall structure,^[^ ^31^ ^],^ and relatively low cost for large‐scale applications.^[^ ^32^ ^]^ These materials and their derivatives have many applications, including catalysis,^[^ ^33^ ^]^ energy storage^[^ ^34^ ^]^ and environmental requalification.^[^ ^35^, ^36^, ^37^ ^]^ Although rare, some PBAs have been used as photocatalysts for degrading organic pollutants as well.^[^ ^38^, ^39^, ^40^ ^]^

In this work, we describe a simple and yet effective synthetic protocol, based on an improved and highly reproducible co‐precipitation method, to obtain nickel hexacyanoferrate (Ni‐HCF) nanocubes, which can be employed as suitable photocatalyst candidate for large‐scale wastewater treatment. The optical and electrochemical properties of Ni‐HCF nanocubes were investigated and tests concerning its photocatalytic activity were carried out to assess the feasibility of MDZ photodegradation. Our findings showed that in aqueous solutions under simulated solar light, a 94.3% degradation of the target contaminant can be attained after 6 h, as the best‐optimized result. We believe that this novel material might offer a safe, economical, eco‐friendly, and scalable solution to remove MDZ and other anthropogenic pollutants from contaminated water.

## 2 Results and Discussion

### 2.1 Structure, Chemical Composition, Optical and Morphological Characterizations

**Figure 1:**
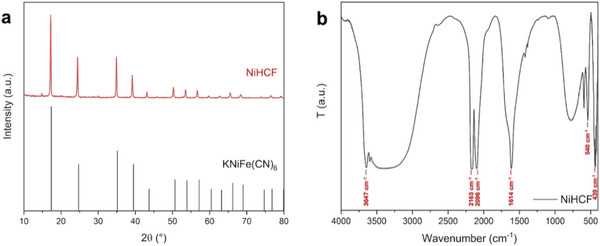
a) XRD patterns of Ni‐HCF nanocubes and reference pattern for potassium nickel iron cyanide (PDF#51‐1897). b) FT‐IR spectrum of Ni‐HCF nanocubes.

The synthetic protocol described in the experimental section allowed obtaining Ni‐HCF nanocubes by co‐precipitation of Ni(OCOCH_3_)_2_⋅4H_2_O and K_3_Fe(CN)_6_. From a structural point of view, the Ni‐HCF sample matches the pattern of potassium nickel iron cyanide, showing the characteristic peaks of this phase as displayed in **Figure** **1**a, in agreement with the values in the standard card (database PDF 51–1897). These peaks correspond to diffraction from the (1 0 0), (1 1 0), (2 0 0), (2 1 0), (2 1 1), (2 2 0), (3 0 0), (3 1 0), (3 2 0), (3 2 1), (4 1 0), and (3 3 0) planes of potassium nickel iron cyanide, respectively. X‐ray diffraction (XRD) pattern confirmed that pure Ni‐HCF presenting a cubic structure can be obtained, thus demonstrating that the proposed strategy meets the requirements for fast and reproducible reactions.

The FT‐IR spectrum of the Ni‐HCF sample, shown in Figure 1b, is characterized by the presence of two major absorption peaks due to the presence of CN groups and water molecules. The absorption band over the range of 2900–3750 cm^−1^ suggests the presence of ─OH groups. The presence of a sharp peak at 3647 cm^−1^ can be attributed to strongly hydrogen‐bonded water molecules in the structure, as suggested by previous works found in the literature.^[^ ^41^ ^]^ The sharp peak at 2165 cm^−1^, instead, corresponds to Fe^III^‐CN‐Ni^II^ linkages. Another less intense peak at 2096 cm^−1^ can be presumably ascribed to unreacted potassium ferricyanide. Whereas in the range between 439 and 540 cm^−1^ the presence of Ni‐C stretching and Ni‐CN bending, respectively, is observed. Moreover, the presence of adsorbed water molecules onto the framework of Ni‐HCF was confirmed by the band at 1614 cm^−1^.

**Figure 2:**
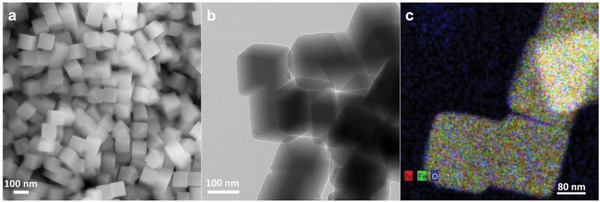
a) SEM micrograph of Ni‐HCF nanocubes. b) TEM micrograph of Ni‐HCF nanocubes. c) EDX mapping showing the distribution of the elements in Ni‐HCF nanocubes. EDX analysis shows the uniform distribution of nickel, iron, and oxygen.

The Ni‐HCF nanocubes were characterized by scanning electron microscopy (SEM) and the micrograph of the sample is shown in **Figure** **2**a. Ni‐HCF appears as perfectly shaped nanocubes with a smooth surface and a rather uniform distribution in size. As a matter of fact, the nanostructures present an average edge size of 190 nm. The comparison of lateral dimensions of the nanostructures to those reported in the literature,^[^ ^42^, ^43^ ^]^ indicates that the compounds produced in this study have a suitable size to be effectively employed in the development of photocatalysts.

High‐resolution transmission electron microscopy (HRTEM) confirmed the SEM analysis, providing that the proposed synthetic protocol yields cubic‐shaped nanostructures (Figure 2b). Moreover, energy dispersion X‐ray (EDX) analysis highlights the presence of oxygen, nickel, and iron (Figure 2c and **Table** **1**), confirming that homogeneous structures were obtained successfully.

**Table 1:** Summary of EDX analysis of Ni‐HCF sample reporting the atomic percentage of oxygen, nickel, and iron elements.

Sample	Element	At%
Ni‐HCF	O	8.7
	Ni	53.1
	Fe	38.2

The UV–vis‐NIR diffuse reflectance spectrum of the nanocubes displayed in Figure S1a (Supporting Information) was recorded to study the optical properties of the compound. The Kubelka–Munk transformation was performed to quantitatively investigate the bandgap of the sample. Considering that Ni‐HCFs show an indirect bandgap, the extrapolation of the bandgap was achieved by plotting (αhν)^1/2^ versus the photon energy, where *α* represents the absorption coefficient, *h* the Plank's constant, and *ν* the frequency. According to the optical data, Ni‐HCF nanocubes show a minimum of reflectance (thus a maximum of absorbance) at ≈400 nm. Figure S1b (Supporting Information) shows the Tauc plot and allows us to extrapolate a bandgap value of 2.34 eV. The nanostructures exhibit a maximum absorbance value that closely aligns with the typical absorption characteristics of MDZ. This feature, along with the determined bandgap, makes Ni‐HCF nanocubes well‐suited for the photo‐degradation of the investigated drug.

**Figure 3:**
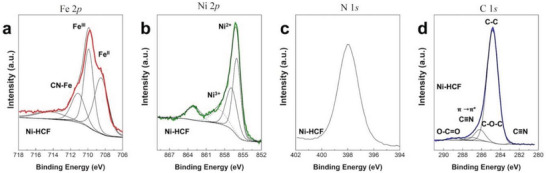
XPS spectra of Ni‐HCF: a) Fe 2*p*, b) Ni 2*p*, c) N 1*s*, and d) C 1*s*.

The chemical composition of Ni‐HCF nanocubes was further characterized with X‐ray photoemission spectroscopy (XPS). The energy of the electronic states of the elements is shown in the high‐resolution spectra of Fe and Ni, as displayed in **Figure** **3**a,b. The spectra of both C 1*s* and N 1*s* (Figure 3c,d) confirm the existence of the cyano‐group in Ni‐HCF. The C 1*s* spectrum presents four contributions with binding energy (BE) of 284.8, 285.3, 286.9, and 288.8 eV, corresponding to the C─C, C─O─, C = O/π→π* transition in the cyanide ligand (C≡N) and O─C = O, respectively.^[^ ^44^ ^]^ Moreover, the contribution at 283.5 eV is lower than that reported for the CN group in K_4_[Fe(CN)_6_]·3H_2_O and K_3_[Fe(CN)_6_] compounds, which appears at 284.4 eV,^[^ ^45^ ^]^ due to the presence of nickel outer metal bonded to CN and alters the electronic coordination of carbon in this compound. The high‐resolution spectrum of N 1*s* presents one peak with a BE of 397.8 eV corresponding to the cyano‐group (C≡N).^[^ ^44^ ^]^ Fe 2*p* signal showed three doublets and the binding energy of the Fe 2*p*_3/2_ photoelectron contributions associated with Fe^2+^ (708.4 eV), Fe^3+^(710.1 eV), and the charge transfer from the C≡N group to the iron (712.3 eV) agree with the values reported in the literature.^[^ ^46^ ^]^ The Ni 2*p* signal of Ni‐HCF shows five contributions with the 2*p*_3/2_ components centered at 856.0, 857.6, and 860.0 eV and assigned in literature to Ni^2+^, Ni^3+^, vibrational satellites.^[^ ^47^ ^]^ The resulting Ni^2+^/Ni^3+^ ratio is 2.7 for the sample. All the data including the surface atomic composition, and related percentages are included in **Table** **2**.

**Table 2:** Atomic concentration (in percentage) derived from XPS analysis.

Sample	C 1*s*	O 1*s*	N 1*s*	Fe 2*p*	Ni 2*p*
Ni‐HCF	50.5	24.8	12.8	4.6	7.1

### 2.2 Evaluation of MDZ Photodegradation Ability Using Ni‐HCF

**Figure 4:**
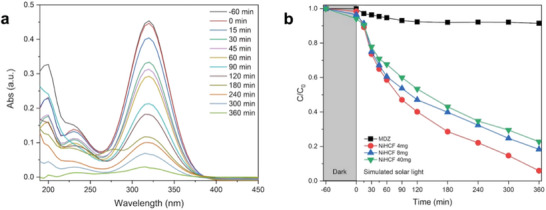
a) Variation of the MDZ absorption spectra during a single photodegradation test under simulated solar light in the presence of 0.05 g L^−1^ Ni‐HCF. b) Photodegradation of MDZ under simulated solar light at room temperature and atmospheric pressure, which is characterized by different loadings of photocatalyst (red, blue, and green curves). Photodegradation of MDZ without the photocatalyst, i.e., the blank, is also reported (black curve). The results are the average of three tests carried out for each photocatalyst loading.

The photodegradation ability of Ni‐HCFs was evaluated under simulated solar light irradiation in aqueous solution at room temperature. For all the tests reported herein, the typical main absorbance peak of MDZ located at ≈320 nm was monitored for 360 min. As shown in **Figure** **4**a, the peak intensity decreases continuously during the observation timelapse. Moreover, Figure 4b displays the photocatalytic degradation of MDZ in terms of the ratio C/C_0_, where C is the MDZ concentration at a given exposure time with respect to its initial value C_0_. Experimentally, the system was left in the darkness for 60 min, thus allowing MDZ to be adsorbed on the surface of the photocatalysts to reach a thermodynamic equilibrium before solar light exposure. As also reported in Figure 4b, the photocatalytic degradation of MDZ was conducted with (red, blue, and green curves) and without (black curves) the photocatalyst. The latter one, which can be regarded as a photolysis experiment used as the reference, shows that the C/C_0_ ratio only slightly decreases (≈8%) over 360 min irradiation time, thus confirming the absence of substantial MDZ photolysis throughout the whole photocatalytic process. This suggests that MDZ has a high physicochemical stability, making it a suitable target model for estimating photocatalytic activity. After 60 min of equilibration in the darkness and in the presence of different loadings of Ni‐HCF samples, a more significant drop in MDZ concentration (compared to its initial concentration) was observed in the samples with a higher dosage. This is justified by the fact that at higher dosages of Ni‐HCF, the phenomenon of MDZ adsorption on the catalyst surface is more pronounced. The adsorption of MDZ on the surface is responsible for a decrease of 2% from the initial MDZ concentration with respect to 0.05 g L^−1^ loading, 4% for 0.1 g L^−1^ loading, and 6% for 0.5 g L^−1^ loading. It is worth mentioning that the efficiency of photocatalytic degradation is highly dependent on the type of adsorption phenomena involved.^[^ ^48^ ^]^ As a matter of fact, the photocatalytic performances of the nanocubes described in this study are closely related to their structure.

The basic Prussian blue material has a face‐centered cubic phase.^[^ ^49^ ^]^ Within this arrangement, Fe^2+^ ions are encompassed by carbon atoms, while Fe^3+^ ions are surrounded by nitrogen atoms, leading to the formation of octahedral structures. To maintain electrical neutrality, the ratio between Fe^2+^ and Fe^3+^ ions should be maintained at ≈3:4. As a result, there are ≈25% vacancies in the [Fe(CN)_6_]_4_^−^ sites that will be filled by H_2_O molecules. In this respect, two types of structural water molecules can be present: i) water in the empty nitrogen sites, where six water molecules coordinate to Fe(III); ii) interstitial water (up to a maximum of eight molecules), which can fill in the center of the unit cell or bind through hydrogen bonds to the coordinated ones. The advantage of the cubic nanostructure lies in enhancing interactions between water molecules and the catalyst's surface, particularly in photocatalysis. With a greater surface area and more exposed active sites than other structures, it facilitates a higher number of crucial interactions. Notably, its ability to accommodate up to 14 water molecules within a Prussian blue unit cell can affect the behavior catalyst, improving its reactivity, and interactions with target molecules.^[^ ^50^ ^]^

This behavior explains the marked decrease of C/C_0_ as a function of the irradiation time when Ni‐HCF samples were added to the solution. The best‐performing loading is 0.05 g L^−1^ (Figure 4b, in red), which degrades up to 94.3% of MDZ within 6 h. Interestingly, the highest loading content of Ni‐HCF (0.5 g L^−1^, Figure 4b, in green) showed the lowest photocatalytic activity (77.4%), while a loading of 0.1 g L^−1^ (Figure 4b, in blue) yields to 81.9% MDZ degradation. When dealing with different catalyst concentrations, the correlation between the physicochemical properties and the photocatalytic performances is often complicated and might lead to conflicting results. It is well known that the amount of catalyst can significantly change the degradation efficiency of an organic pollutant. Moreover, a higher amount of catalyst is not always a sign of better photocatalytic efficiency. In fact, when the catalyst concentration is very high, an inhibition of the photocatalytic activity takes often place. Eventually, the results of the present work are no exception. The inhibition can be ascribed to the aggregation of Ni‐HCF nanocomposite in the reactor, which reduces the number of available sites involved in the photocatalytic reaction.^[^ ^50^ ^]^ Besides that, an excessive dosage of catalyst can contribute to an increase in turbidity and opacity of the solution, which can hamper the light absorption on the surface of the catalyst.^[^ ^51^, ^52^ ^]^ More details concerning the kinetic constants evaluated during the photodegradation experiments are provided in the Supporting Information (Figures S2 and S3, Table S1, Supporting Information). The photocatalytic activity of the best‐performing loading (0.05 g L^−1^) was also evaluated under both UV and visible light, in the same experimental conditions used before, to investigate any wavelength dependence. As a result, Ni‐HCF photocatalyst provided an MDZ degradation of 94.4% and 46.0% under UV and visible light, respectively, after 180 min irradiation time (Figures S2 and S3, Supporting Information).

### 2.3 Recycling the Catalyst

The recyclability of a catalyst is an important parameter for its practical use. Therefore, stability and reusability tests were carried out for the best‐performing loading (0.05 g L^−1^) of photocatalyst under simulated solar light irradiation in the same experimental conditions, as shown in Figure S4 (Supporting Information). Although a slight decrease of the photocatalytic activity (−3.2%) was observed soon after the first‐cycle test, the performance was restored on average after the third cycle where a photodegradation of 91.4% was achieved, in line with what was obtained in the previous tests. Moreover, a minimal increase of the MDZ adsorption on the photocatalyst surface was recorded in the darkness timelapse, which ranges from 4.5% of the first cycle to 4.9% of the third one. This phenomenon was attributed to both the presence of MDZ molecules and degradation by‐products adsorbed by previous cycles onto the active sites, resulting in a reduced rate of radicals' production. An FT‐IR analysis on the photocatalyst before photodegradation and after three catalytic cycles showed no variations in the overall structure of the nanomaterial, as summarized in Figure S5 (Supporting Information) and supported by the functional tests displayed in Figure S6 (Supporting Information). In fact, the latter showed no significant variation in the photocatalytic activity after three cycles of reusability tests. We can therefore confidently state that Ni‐HCF is not only still active toward MDZ degradation under simulated solar light, but it provides very good physicochemical stability, thus supporting its long‐term usage.

### 2.4 Proposed Mechanism

**Figure 5:**
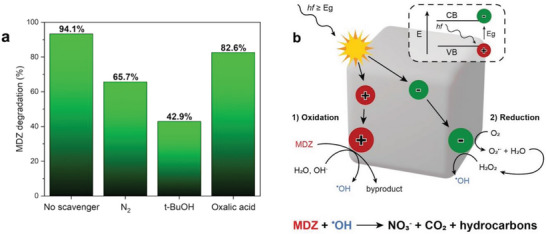
a) Role of radical scavengers on the MDZ photodegradation with Ni‐HCF. b) Proposed photodegradation pathway of MDZ by means of Ni‐HCF under simulated solar light irradiation at room temperature and atmospheric pressure.

To investigate the main active species involved in the MDZ degradation, free radical trapping experiments were carried out, which considered hydroxyl radical (^•^OH), holes (h^+^), and superoxide (^•^O_2_^¯^). As shown in **Figure** **5**a, the photocatalytic efficiency of Ni‐HCF was significantly inhibited when *t*‐BuOH, a well‐known scavenger for ^•^OH, was introduced into the system.

Whereas it was barely affected by the addition of oxalic acid, a well‐known scavenger for h^+^. Instead, a slight decrease in the photocatalytic activity is observed upon flushing N_2_, which is used as a scavenger for O_2_^¯^. Hence, hydroxyl and superoxide radicals should be the two major reactive species involved in the MDZ degradation by Ni‐HCF under simulated solar light irradiation. Upon these considerations, we propose a plausible mechanism for photodegradation as summarized in Figure 5b.

### 2.5 Electrochemical Characterization

**Figure 6:**
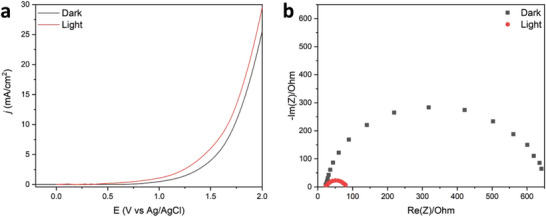
Electrochemical characterization of Ni‐HCF nanocubes in 1 m KOH in the darkness (black curve) and under simulated solar light irradiation (red curve). a) LSV was carried out at a scan rate of 50 mV s^−1^. b) Nyquist plot obtained from EIS measurements showing the resistance to charge transfer of Nyquist plot for the experiment carried under solar simulated light.

To provide further details on the photocatalytic properties of Ni‐HCF nanocubes, an electrochemical characterization was performed. The linear sweep voltammetry (LSV) plot, reported in **Figure** **6**a, showed that the solvent/electrolyte discharge is anticipated at ≈118 mV under solar light irradiation (red curve) when compared to the darkness (black curve). Whereas the current delivery at +1.5 V versus Ag/AgCl is almost double when the sample is exposed to solar light irradiation.

Furthermore, to investigate the interfacial charge transfer processes, electrochemical impedance spectroscopy (EIS) was employed, and the results are summarized in Figure 6b and Figure S7 (Supporting Information). In general, a small radius in EIS Nyquist plots implies the rapid separation and transfer of photoexcited electron‐hole pairs. It is clear from Figure 6b that the solar light irradiation of the catalysts remarkably reduced the arc radius, indicating lower interface charge transfer resistance.

As a matter of fact, the fitting of the experimental data yielded a simplified model for the Randles circuit, reported in Scheme S1 (Supporting Information), composed of two resistors and a capacitor, whose calculated values are reported in Table S2 (Supporting Information). Indeed, the resistance of the solution (R_s_) is comparable in the two different experimental conditions (33.15 and 36.65 Ω for darkness and solar light irradiation, respectively), which implies that the difference in the overall behavior is not related to the interactions between the electrode and the solvent/electrolyte. On the other hand, the difference in resistance toward the charge transfer is remarkable. Under solar‐simulated light, the sample decreases its charge transfer resistance (R_ct_) by ≈8 times (625.1 and 81.35 Ω for darkness and solar light irradiation, respectively). This evidence witnesses that, upon solar light irradiation, Ni‐HCF nanocubes possess a higher conductivity, which leads to a fast charge transfer and, hence, enhanced photodegradation.

In **Table** **3**, the performances of different nanostructures toward the photodegradation of MDZ are listed as reported in the literature^[^ ^9^, ^25^, ^53^, ^54^, ^55^, ^56^, ^57^ ^]^ and compared to that of the Ni‐HCF nanocubes investigated in this study. The comparison highlights that our Ni‐HCF nanocubes offer the possibility to exploit UV, visible, and simulated solar light while providing good to excellent performances for MDZ photodegradation. Instead, other photocatalysts might perform well under one range of wavelengths but not under all of them. The ability of Ni‐HCF to perform well under UV, visible, and simulated solar light indicates its versatility and potential use.

**Table 3:** Comparison of the performances of different nanostructures toward the photodegradation of MDZ as reported in the literature and compared to Ni‐HCF nanocubes investigated in this study.

Material	Catalyst dosage [g L^−1^]	Initial concentration [mg L^−1^]	Irradiation source	Time [min]	Removal efficiency [%]	Reference
BiVO_4_/BiPO_4_	0.5	5	UV	120	75.5	[53]
BiVO_4_/BiPO_4_	0.5	5	Visible	360	64.5	[53]
BiCN_3_	1	5	Visible	180	95.0	[54]
CuBi_2_O_4_/CuO	2	50	Visible	120	36.0	[9]
ZnO/RGO	1	5	Visible	160	49.3	[25]
P‐doped g‐C_3_N_4_/Co_3_O_4_	1	10	Visible	180	70.0	[55]
BiVO_4_/FeVO_4_	4	10	Visible	90	91.0	[56]
AgI/ZnO	1	2	Simulated sunlight	30	67.1	[57]
Ni‐HCF	0.05	8.6	UV	180	94.4	This work
Ni‐HCF	0.05	8.6	Visible	180	46.0	This work
Ni‐HCF	0.05	8.6	Simulated sunlight	360	94.1	This work

Moreover, the references reported in Table 3 highlight the significance of a study regarding the photodegradation of MDZ, which has not been extensively investigated, as indicated by the limited number of publications on the topic. The difficulty in breaking down this persistent molecule also made it a challenging subject to investigate. It is also worth noting that no cubic shape has been reported in the literature to date, which makes this study even more relevant. Additionally, the use of simulated solar light, except for one prior study,^[^ ^57^ ^]^ has never been utilized in previous research. The use of simulated solar light can indeed provide several advantages. For instance, it can eliminate the need for energy‐demanding UV lamps, which aligns with the principles of sustainable development. This can contribute to reducing the environmental impact of scientific research and real‐world applications, therefore making it more sustainable.

## 3 Conclusion

In the past years, conventional biological and physical treatment methods have been the mainstream techniques to remove organic pollutants from wastewater. Nevertheless, the decontamination of many emerging anthropogenic organic pollutants requires novel techniques and materials capable of removing them rapidly and safely. Ideally, they also should be inexpensive and environmentally safe, meaning that the reagents, the energy source, and the catalysts themselves should be abundant, cheap, and environmentally friendly. We have described a simple and yet effective synthetic protocol, based on a co‐precipitation method, to obtain pure Ni‐HCF nanocubes, a Prussian blue derivative, which were fully characterized and showed a well‐defined, pure crystalline structure and enhanced photocatalytic and electrochemical properties. The electrochemical characterizations also pointed out that, when irradiated with simulated solar light, the material was able to deliver higher current densities with a remarkable drop in the resistance toward the charge transfer. This suggested an effective electron‐hole separation, thus justifying the enhanced photocatalytic performances. Indeed, we investigated the photodegradation of MDZ, a persistent antibiotic that is found in many water sources and is difficult to break down. Therefore, MDZ can be regarded as a reference molecule for such application. Our findings showed that, depending on the loading of photocatalyst in solutions, the photodegradation of MDZ varies between 77.4% and 94.3%, thus witnessing the excellent performance of the catalyst for such applications without using co‐catalysts and/or additives such as H_2_O_2_. The catalyst has been further studied in terms of stability and recycling, showing remarkable performances even after three cycles of reuse, a key parameter for possible future water remediation applications. Moreover, the scavenger tests also evidence what active radicals are involved, suggesting a photodegradation mechanism in which the main role is played by hydroxyl radicals. We believe that the results presented in this study will be extremely useful in promoting the development of efficient photocatalysts with broad‐band solar spectrum absorption (maximizing the use of solar energy radiation) which might offer a safe, economical, and eco‐friendly solution for the treatment of contaminated water on a large scale.

## 4 Experimental Section

### Synthesis

Ni‐HCF nanocubes were prepared by a co‐precipitation method. Briefly, 1.40 g of Ni(OCOCH_3_)_2_⋅4H_2_O (5.5 mmol) and 3.30 g of sodium citrate dihydrate (11 mmol) were dissolved in 200 mL of deionized water, leading to solution A. In the meanwhile, 1.30 g of K_3_Fe(CN)_6_ (4 mmol) were dissolved into 200 mL of deionized water, leading to solution B. The solutions A and B were then mixed at room temperature under continuous stirring for 24 h. Once a light‐brown suspension was obtained, the reaction was stopped. Eventually, the crude was centrifuged (5000 rpm for 10 min), washed with deionized water, and dried at 65 °C overnight. Upon conducting this synthesis, the catalyst was obtained with a substantial yield of 3.58 grams (0.895 g per 100 mL of distilled water), showcasing the scalability of the approach.

## Conflict of Interest

The authors declare no conflict of interest.

## Supporting information

Supporting Information

## Data Availability

The data that support the findings of this study are available from the corresponding author upon reasonable request.
