# Supplementary material for: Highly Efficient Solar‐Light‐Driven Photodegradation of Metronidazole by Nickel Hexacyanoferrate Nanocubes Showing Enhanced Catalytic Performances
Source: Small Methods. 2024 Feb 17;9(2):2301541. doi: 10.1002/smtd.202301541 (PMC11843405; doi:10.1002/smtd.202301541)
Supplement: Supplementary file 1 — Supporting Information [file SMTD-9-2301541-s001.pdf]

small
methods

Supporting Information

for *Small Methods*, DOI 10.1002/smtd.202301541

Highly Efficient Solar-Light-Driven Photodegradation of Metronidazole by Nickel Hexacyanoferrate Nanocubes Showing Enhanced Catalytic Performances

Edlind Lushaj, Matteo Bordin, Kamran Akbar, Letizia Liccardo, Isabel Barroso-Martín, Enrique Rodríguez-Castellón, Alberto Vomiero, Elisa Moretti* and Federico Polo**

Supporting Information

Highly efficient solar-light-driven photodegradation of metronidazole by nickel hexacyanoferrate nanocubes showing enhanced catalytic performances

Edlind Lushaj, Kamran Akhbar, Matteo Bordin, Letizia Liccardo, Isabel Barroso-Martin, Enrique Rodríguez-Castellón, Alberto Vomiero, Elisa Moretti*, Federico Polo**

Experimental

Chemicals. Nickel(II) acetate tetrahydrate $Ni(OCOCH_3)_2{\cdot}4H_2O$ (98%), trisodium citrate dihydrate $HOC(COONa)(CH_2COONa)_2{\cdot}2H_2O$ (≥99.0%), potassium ferricyanide $K_3Fe(CN)_6$ (≥99.0%), and absolute ethanol (99%) were purchased from Sigma-Aldrich (Merck Group) and used as received for the preparation of all the samples.

Material characterization. The crystallographic structure, morphology, composition, optical and electrochemical properties of the samples were determined as it follows.

X-ray diffraction. The crystallographic structure of the samples was determined by X-ray diffraction (XRD). XRD patterns were collected using an X-ray diffractometer (PanAnalytical Empyrean XRD) with Cu Kα radiation in the range 2θ between 10° and 80°.

Scanning and transmission electron microscopies. The morphology, composition and elemental mappings were obtained by a field-emission scanning electron microscopy (FESEM), Magellan XHR 400L with a 5 kV electron beam. TALOS F200x equipment working both in high-resolution transmission electron microscopy (HRTEM) and scanning transmission electron microscopy (STEM) modes was used to obtain HRTEM images to analyze the morphology. Microanalysis was performed using an energy dispersion X-ray spectrometer (EDX) Super-X system with 4 X-Ray detectors and an X-FEG beam.

X-ray photoelectron spectroscopy. The chemical composition was determined by X-ray photoelectron spectroscopy (XPS) measurements were recorded by The Scienta ESCA 200 Spectrometer operating with monochromatic X-ray source Al(k-alpha) of photons at 1486.6 eV under ultra-high vacuum and low pressure of 10^{-10} mbar. The experimental methodology of XPS contained 0.65 eV Au $4f_{7/2}$ line of full width at half maximum. A Multipak-V9.3

software package was used for data analysis. A Shirley-type background was subtracted from the signals, and the recorded spectra were always fit using Gaussian−Lorentzian curves to determine more accurately the binding energy of the different element core levels.

Infrared absorption spectroscopy. FT-IR spectra were collected in the range 4000-400 cm^{-1} using a Perkin Elmer FT-IR-spectrometer Spectrum One equipped with a holder for transmission measurement with adapter to accommodate the KBr pellets.

Reflectance spectroscopy. UV-Visible-NIR reflectance spectra was recorded to study the optical properties of the compound. The data were collected with a Perkin-Elmer UV-Vis-NIR Lambda 1050+ spectrometer between 200 and 1200 nm. The Kubelka-Munk transformation was performed to quantitatively investigate the band gap of the sample. The results are displayed in **Figure S1**.

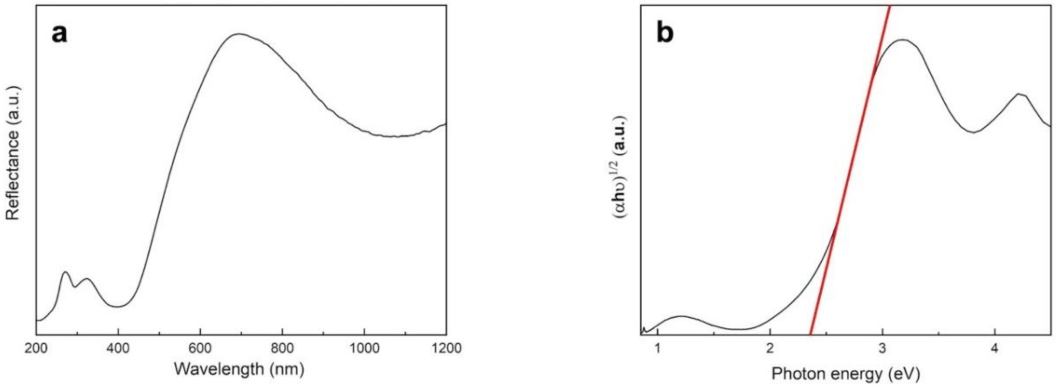

Figure S1. (a) Reflectance curve of Ni-HCF nanocubes and (b) corresponding Kubelka-Munk transformation.

Electrochemistry. The ability of the sample to separate the photogenerated charge carriers was investigated by means of an electrochemical workstation (BioLogic SP-300, equipped with EIS module) in a three-electrode configuration system at room temperature. A glassy carbon electrode (GCE) with a diameter of 3.0 mm, Ag/AgCl (3 M KCl), and a Pt wire were used as working, reference, and counter electrodes, respectively. Linear sweep voltammetry (LSV) was performed in 1.0 M KOH electrolyte in the dark and under simulated solar light. The catalyst was prepared by dispersing 5 mg of Ni-HCF powder in 20 μL Nafion (5 wt. % in lower aliphatic alcohols and water, contains 15-20% water, Sigma-Aldrich) and 200 μL isopropyl alcohol (99.5%, Sigma-Aldrich) solution, and stirring for 1 hour. Then, the working

electrode was prepared by dropping 5 μL of the catalyst ink on the GCE. Electrochemical impedance spectroscopy (EIS) was used to investigate interfacial charge transfer process and the analysis was carried out over the frequency range 100 kHz-100 mHz at an applied potential of 0.65 V with an AC amplitude of 10 mV.

Kinetics and photodegradation under different light irradiation

MDZ was chosen as a test molecule to evaluate the photocatalytic activity of as-synthesized samples under simulated solar light irradiation. The initial concentration of the target molecule was set to be 5.0×10^{-5} M at pH = 6.3. All the photodegradation tests were carried out at 20 °C. In addition to this, different tests were conducted using a variable amount of catalyst (0.05 g L^{-1}, 0.1 g L^{-1} and 0.5 g L^{-1}) to optimize the concentration of the catalyst in solution. Under simulated solar light irradiation, an ABET Solar Simulator with an AM1.5G filter and a 100W xenon arc lamp was used. Before each measurement, the intensity of the light source was set using a reference cell to standardize the illumination conditions. The photocatalytic activities of the samples were further evaluated under UV light using a 125 W high pressure mercury lamp, operating at wavelengths between 180 and 420 nm with a peak at 366 nm and the sample was placed right underneath the lamp's filament at a distance from the glass surface of 10 cm. The photodegradation experiments under Visible light were carried out using an ABET Solar Simulator with an AM1.5G filter and a 100W xenon arc lamp equipped with a 400 nm cutoff filter. Prior to each type of irradiation, the suspension was kept under stirring in dark conditions for 60 minutes to allow the system to reach the adsorption equilibrium. After exposing the solution to light, aliquots of about 1 mL of the solution containing both the target molecule and the catalyst were collected. Each aliquot was filtered through a 0.45 μm PTFE Millipore disc to remove the catalyst. A Perkin-Elmer UV-Vis-NIR Lambda 1050+ spectrometer was used to quantify the drug concentration. The degradation processes were monitored following the variations in absorbance of the maximum of the UV-Vis spectra of the target molecule (320 nm). Once the concentration at 320 nm was extrapolated using the Lambert-Beer law, it was possible to evaluate the degradation rate C/C_0, where C is the concentration after time t and C_0 represents the initial concentration at t = 0. Kinetics studies regarding the photodegradation of MDZ molecules were carried out evaluating the 0-45 min data interval and employing the pseudo-first-order model:

$$\ln(C/C_0) = -kt$$

where k is the pseudo-first-order rate constant (min^{-1}), calculated as:

k = 2.303 × slope

The results are shown in **Figure S2** and **S3**, whereas the estimated kinetic constants are reported in **Table S1**.

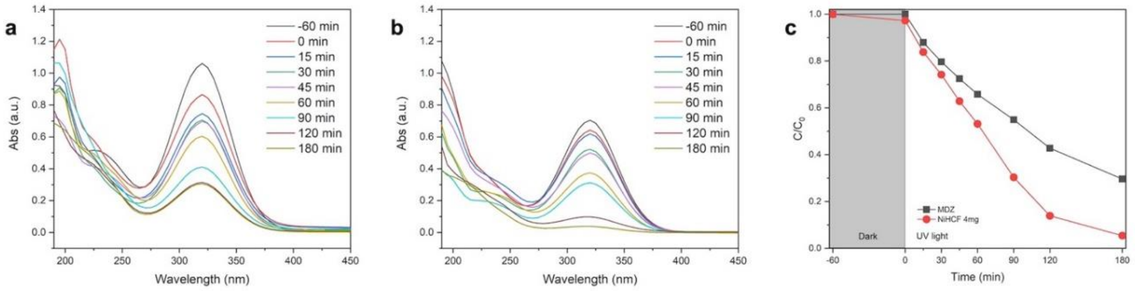

Figure S2. Absorption spectra of MDZ photodegradation under UV light irradiation (a) without photocatalyst and (b) with 0.05 g L^{-1} of Ni-HCF photocatalyst. (c) Photodegradation curves of MDZ under UV light with (red curve) and without (black curve) the Ni-HCF photocatalyst. All the experiments were carried out at room temperature and atmospheric pressure with an irradiation of 180 min.

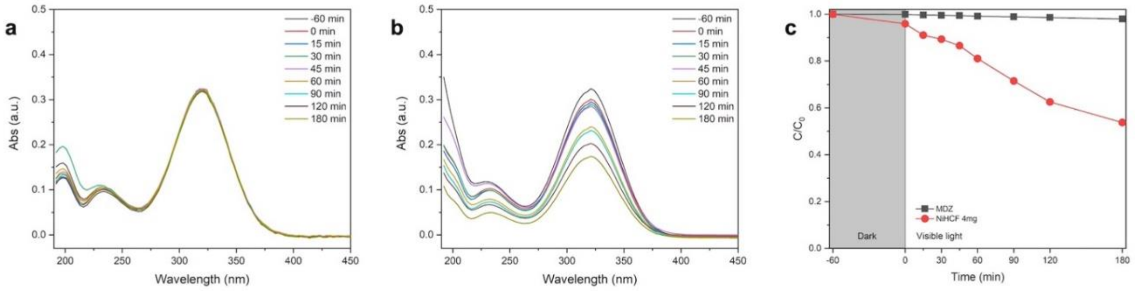

Figure S3. Absorption spectra of MDZ photodegradation under visible light irradiation (a) without photocatalyst and (b) with 0.05 g L^{-1} of Ni-HCF photocatalyst. (c) Photodegradation curves of MDZ under visible light with (red curve) and without (black curve) the Ni-HCF photocatalyst. All the experiments were carried out at room temperature and atmospheric pressure with an irradiation of 180 min.

Table S1. Kinetic constant values for the photodegradation of MDZ as is and for MDZ with different loadings of photocatalyst under simulated solar light (SSL).

Sample	k SSL (min^{-1})
MDZ	$2.74 \cdot 10^{-3}$
Ni-HCF (4 mg)	$22.32 \cdot 10^{-3}$
Ni-HCF (8 mg)	$21.33 \cdot 10^{-3}$
Ni-HCF (40 mg)	$17.55 \cdot 10^{-3}$

Recycling of the catalyst

A 3-cycle recycling test was performed to evaluate the stability and reusability of the photocatalyst. The catalyst was recovered after each photocatalytic cycle by centrifugation. The solution containing the material was placed in 2 mL cuvettes and subsequently centrifuged at 10000 rpm for 3 minutes. The supernatant was removed, and the remaining powder was washed several times with deionized water and ethanol to remove possible traces of the antibiotic. After washing, the sample was dried overnight at 65 °C. Then the recovered dried powder was redispersed in the investigated solution and the next photocatalytic cycle was carried out, following the same procedure as described above.

The main active species involved in the MDZ degradation pathway were investigated by performing free radical trapping experiments. Briefly, different photocatalytic tests were carried out using the same conditions aforementioned, except for the addition to the reaction system of tert-Butyl alcohol (*t*-BuOH, 1 mM), oxalic acid (1 mM) and N_2 (g) as $^{\bullet}OH$, holes (h^+), and O_2^- scavengers, respectively.

Where specified, each photocatalytic test is reported to be the average of 3 tests repeated in the same conditions. In those cases, uncertainties regarding the photodegradation measurements were not reported in the catalytic profiles since the acquisition accuracy of the UV-Vis spectrophotometer used to obtain the data was 10^{-4}. This does not allow the display of error bars in the graphs that will follow as they are too small and indistinguishable to be reported.

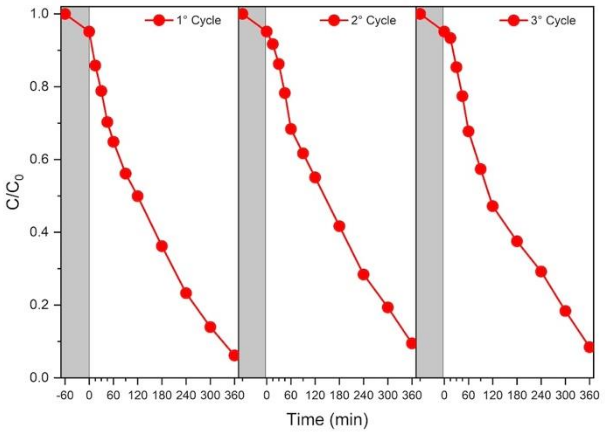

Figure S4. 3-cycles reusability test for the photodegradation of MDZ under simulated solar light in presence of Ni-HCF.

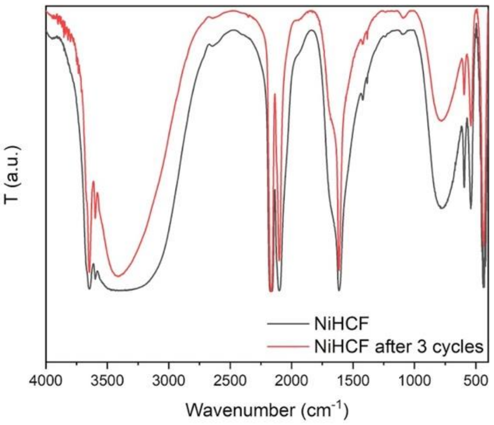

Figure S5. FTIR spectra of Ni-HCF before (black curve) and after 3 cycles (red curve) of MDZ photodegradation experiments.

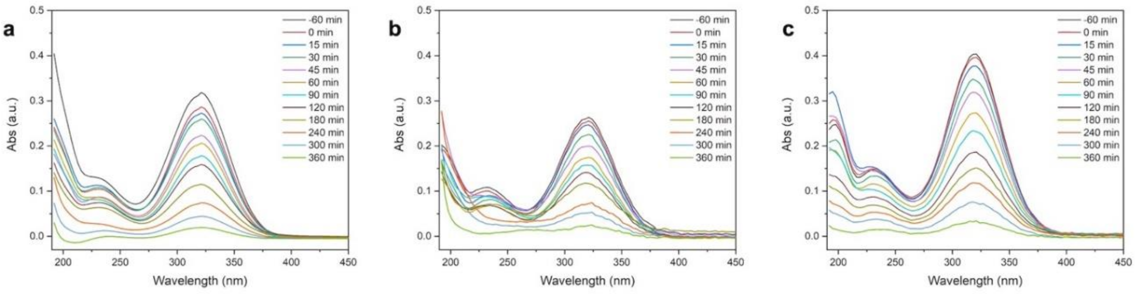

Figure S6. Recycling test showing the absorption spectra of MDZ under simulated solar light irradiation with 0.05 g L^{-1} N-HCF photocatalyst. (a) First, (b) second, and (c) third cycle. All the tests were carried out at room temperature and atmospheric pressure.

Electrochemical Impedance Spectroscopy

To investigate the interfacial charge transfer processes, electrochemical impedance spectroscopy (EIS) was employed, and the results are summarized in Figure 6 in the main text and in the following **Figure S8**.

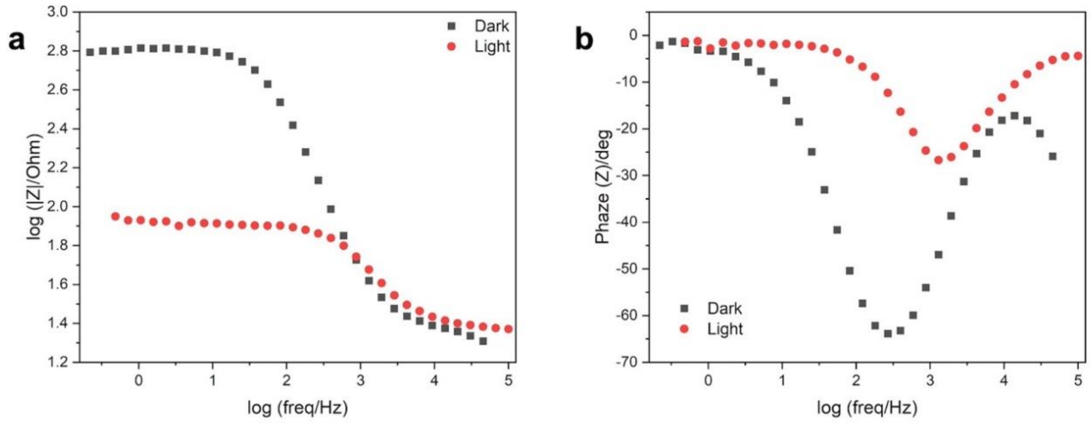

Figure S7. EIS analysis showing (a) the Bode module and (b) the Bode phase plots of Ni-HCF nanocubes in the darkness (black curve) and under simulated solar light irradiation (red curve) recorded in 1 M KOH over the frequency range 100 kHz-100 mHz at an applied potential of 0.65 V with an AC amplitude of 10 mV.

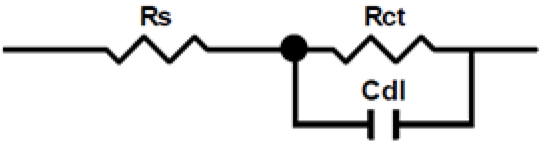

Scheme S1. Equivalent Randles (simplified) circuit.

Table S2. Results obtained upon the fitting of the simplified Randles equivalent circuit[a] displayed in **Scheme S1** for the EIS measurements carried out in the darkness and under solar light irradiation.

	R_s (Ω)	R_{ct} (Ω)	C_{dl} (μF)
Darkness	33.15 ± 0.40	625.1 ± 0.40	4.132 ± 0.014
Light	36.62 ± 0.70	81.35 ± 0.26	1.638 ± 0.054

[a] Equivalent circuit: R_s / (R_{ct} + C_{dl})
